# Deep Transfer Learning for Classification of Late Gadolinium Enhancement Cardiac MRI Images into Myocardial Infarction, Myocarditis, and Healthy Classes: Comparison with Subjective Visual Evaluation

**DOI:** 10.3390/diagnostics15020207

**Published:** 2025-01-17

**Authors:** Amani Ben Khalifa, Manel Mili, Mezri Maatouk, Asma Ben Abdallah, Mabrouk Abdellali, Sofiene Gaied, Azza Ben Ali, Yassir Lahouel, Mohamed Hedi Bedoui, Ahmed Zrig

**Affiliations:** 1 Technology and Medical Imaging Laboratory LR12ES06, Faculty of Medicine of Monastir, University of Monastir, Monastir 5019, Tunisia; 2 Faculty of Sciences of Monastir, University of Monastir, Monastir 5019, Tunisia; 3 LR18-SP08 Department of Radiology, University Hospital of Monastir, Monastir 5019, Tunisia

**Keywords:** deep learning, VGG16, myocardial infarction, myocarditis

## Abstract

**Background/Objectives:** To develop a computer-aided diagnosis (CAD) method for the classification of late gadolinium enhancement (LGE) cardiac MRI images into myocardial infarction (MI), myocarditis, and healthy classes using a fine-tuned VGG16 model hybridized with multi-layer perceptron (MLP) (VGG16-MLP) and assess our model’s performance in comparison to various pre-trained base models and MRI readers. **Methods:** This study included 361 LGE images for MI, 222 for myocarditis, and 254 for the healthy class. The left ventricle was extracted automatically using a U-net segmentation model on LGE images. Fine-tuned VGG16 was performed for feature extraction. A spatial attention mechanism was implemented as a part of the neural network architecture. The MLP architecture was used for the classification. The evaluation metrics were calculated using a separate test set. To compare the VGG16 model’s performance in feature extraction, various pre-trained base models were evaluated: VGG19, DenseNet121, DenseNet201, MobileNet, InceptionV3, and InceptionResNetV2. The Support Vector Machine (SVM) classifier was evaluated and compared to MLP for the classification task. The performance of the VGG16-MLP model was compared with a subjective visual analysis conducted by two blinded independent readers. **Results:** The VGG16-MLP model allowed high-performance differentiation between MI, myocarditis, and healthy LGE cardiac MRI images. It outperformed the other tested models with 96% accuracy, 97% precision, 96% sensitivity, and 96% F1-score. Our model surpassed the accuracy of Reader 1 by 27% and Reader 2 by 17%. **Conclusions:** Our study demonstrated that the VGG16-MLP model permits accurate classification of MI, myocarditis, and healthy LGE cardiac MRI images and could be considered a reliable computer-aided diagnosis approach specifically for radiologists with limited experience in cardiovascular imaging.

## 1. Introduction

Cardiac magnetic resonance imaging (MRI) is an effective method for non-invasively assessing cardiovascular morphology, myocardial perfusion, ventricular function, and tissue characterization [1]. In cardiac MRI, late gadolinium enhancement (LGE) sequences offer a reliable diagnosis of myocardial infarction (MI) and myocarditis due to their ability to detect areas of myocardial necrosis and fibrosis with high spatial resolution [2]. In MI, LGE is typically subendocardial or transmural, located in a region corresponding to the perfusion area of a coronary artery [3]. Meanwhile, myocarditis is associated with epicardial and midmyocardial LGE patterns [4]. In clinical practice, LGE regions are assessed visually, potentially leading to inconsistencies within and between different observers [5]. Furthermore, fine subepicardial enhancement in myocarditis may be misinterpreted as epicardial fat, while fine subendocardial enhancement in MI could be mistaken for the ventricular cavity, which may result in the misclassification of pathological LGE images as normal. Moreover, in cases of quasi-transmural enhancement, distinguishing myocarditis from MI is particularly challenging. Such uncertainty is frequently noted among radiologists or cardiologists with limited experience in cardiovascular imaging. Recently, machine learning has proven to have significant potential in this field [6]. It has been recognized as effective in predicting and detecting various cardiovascular diseases [7,8,9]. According to a recent study, machine learning can help to differentiate between MI and myocarditis with an accuracy of 89% using LGE cardiac images [5]. Deep learning, an advanced subset of machine learning, reveals its potential in several fields, particularly in cardiovascular MRI by aiding clinicians in image analyses, image interpretation, and patient assessment [10]. It has the capability to extract relevant features from images, which is highly valuable to the analysis of medical images. Furthermore, in contrast to machine learning, which requires human intervention for feature extraction, deep learning offers the advantage of automatically identifying and extracting key features in an end-to-end process [11,12]. Deep learning includes a class of algorithms known as neural networks. Notably, convolutional neural networks (CNNs) are currently acknowledged as the state of the art for image prediction [13]. CNNs have been recently implemented to address radiological issues [14] and have demonstrated the ability to classify medical images at a level that rivals or even surpasses that of experts in various fields [15]. A study has demonstrated that CNN networks can effectively categorize subjects with normal, hypertrophic cardiomyopathy and dilated cardiomyopathy on cine MRI images [16]. Another study utilized deep learning for the binary classification of patients with and without cardiac amyloidosis based on clinical and imaging features extracted from LGE images [17]. One of the most popular architectures in CNNs is Visual Geometry Group-16 (VGG16). It demonstrates a considerable capacity for feature extraction, enabling it to achieve significant results in image classification [18]. To avoid inconsistencies between readers and to automate redundant tasks, our study aimed to develop a computer-aided diagnosis (CAD) approach for the classification of LGE cardiac MRI images into MI, myocarditis, and healthy classes using a fine-tuned VGG16 CNN model hybridized with multi-layer perceptron (MLP) (VGG16-MLP) and to assess our model’s performance in comparison to various pre-trained base models and cardiac MRI readers.

## 2. Materials and Methods

### 2.1. Study Population

This retrospective study included LGE images from 163 patients who underwent cardiac MRI between 2015 and 2022 in the Medical Imaging Department. Study selection was made by a 15-year-experienced radiologist specializing in cardiovascular imaging, evaluating and labeling LGE images based on their representative appearance as healthy, MI, and myocarditis. The dataset is described in Table 1.

### 2.2. MRI Acquisitions

All cardiac MRI acquisitions were performed on a Philips Ingenia 1.5 Tesla MRI (Philips Healthcare, Best, The Netherlands) using a torso phased-array coil coupled to a spine coil. A standard cardiac protocol was used, including cine steady-state free precession in short-axis, long-axis, and four-chamber views; a black blood short-time inversion recovery sequence; and an LGE sequence. LGE images were acquired 10 min after the administration of a 0.2 mmol/kg bolus of adulterate dimeglumine (Dotarem, Guerbet, Roissy, France). Short-axis sections of LGE were obtained with a 15 s breath-hold using the following parameters: flip angle, 25°; repetition time, 6 ms; echo time, 3 ms; field of view, 25 cm; slice thickness, 10 mm.

### 2.3. Image Preparation

#### 2.3.1. Extraction of the Region of Interest (ROI): Left Ventricle Segmentation

**Figure 1:**
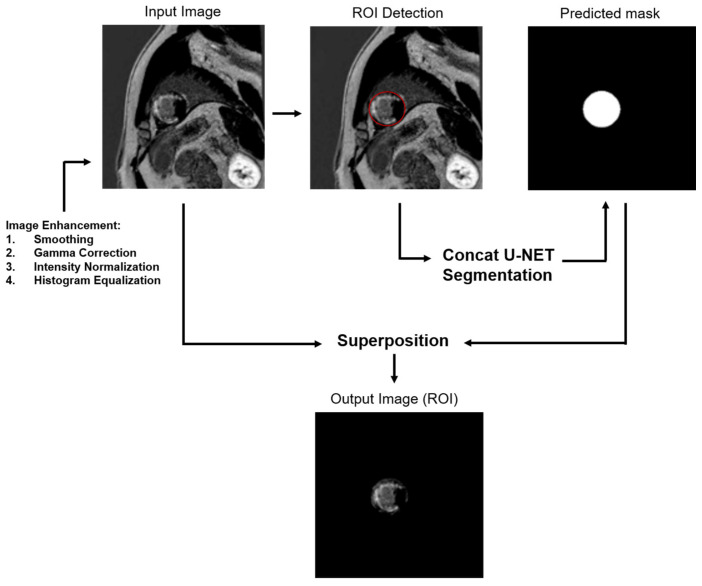
Framework for the extraction of the region of interest (ROI).

A segmentation of the left ventricle, which includes the left myocardium and left ventricle cavity, was performed using a deep learning method developed in our laboratory [19]. The procedure is described in Figure 1.

##### Image Preprocessing for Segmentation

The left ventricle in LGE images was manually segmented to create the ground truth. Four operations were conducted on cardiac LGE images and their corresponding ground truths to enhance image contrast: smoothing, gamma correction, intensity normalization, and histogram equalization. A patch extraction was applied, dividing the image into small patches to increase the effective number of training data. In total, 20% of the data were used for the test.

##### Concat-U-Net Segmentation

For the deep learning segmentation process, Concat-U-Net was employed, harnessing the strengths of three distinct architectures: U-Net, ResNet, and DenseNet [19]. This architecture is similar to U-Net. However, it has the advantage of using fewer parameters to reduce the risk of overfitting.

##### Superposition

The mask was superposed to the original image to obtain an image of the ROI.

#### 2.3.2. Image Resizing

The images were adjusted from the size of (320 × 320) to (224 × 224) in order to meet the requirements of the model.

#### 2.3.3. Data Augmentation

To increase the diversity of the training dataset, geometric data augmentation was applied during training, including a 10% horizontal shift range and a 15% zoom range.

### 2.4. Deep Learning Classification

#### 2.4.1. Model Description

**Figure 2:**
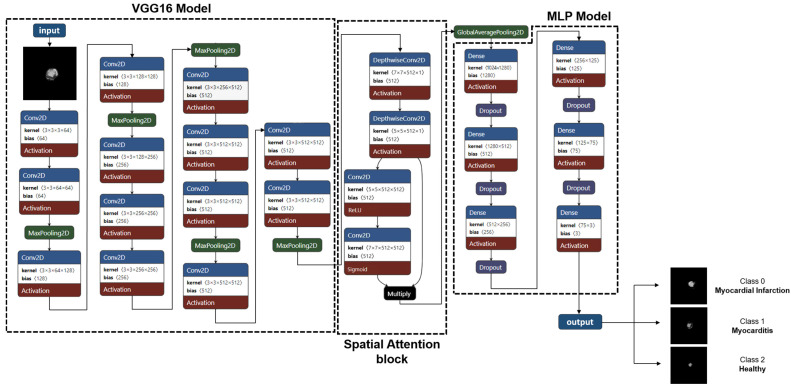
Descriptive architecture of our deep learning model VGG16-MLP composed of fine-tuned Visual Geometry Group (VGG16), spatial attention mechanism, and multi-layer perceptron (MLP).

Our model combined a fine-tuned VGG16 model with MLP. For feature extraction from input images, an ImageNet-pre-trained VGG16 model was performed. All layers in the base model were frozen except for the last 3 layers. This means that the weights of most layers in the base model were not be updated during training. The VGG16 model consists of five blocks of convolutional layers with small 3 × 3 filters, each followed by a max-pooling layer to reduce the spatial dimension. A spatial attention mechanism was implemented to enhance the model’s ability to focus on relevant features. This was achieved using two successive DepthwiseConv2D layers with 7 × 7 and 5 × 5 kernels, capturing multiscale spatial information while significantly reducing the number of parameters and computational cost. These layers were followed by two standard Conv2D layers with ReLU and Sigmoid activation functions, respectively. ReLU was used after the first Conv2D layer to introduce non-linearity while preserving and propagating important spatial features, while Sigmoid activation was applied after the second Conv2D layer to generate attention weights between 0 and 1. This combination enables the model to extract meaningful spatial features and create a normalized attention map that determines which spatial regions to emphasize or suppress when multiplied by the original features. Then, a GlobalAveragePooling2D layer was used to reduce the spatial dimensions of the feature maps into a single vector, which served as input for the MLP. The MLP architecture used for the classification task included five dense layers integrated with dropout for regularization, which formed the network’s latter part, with a final dense layer producing a 3-unit output. The architecture of our model VGG16-MLP is presented in Figure 2.

#### 2.4.2. Model Assessment: Training, Validation, and Test

The training dataset consisted of 669 images (80% of the dataset). After data augmentation using 10% horizontal shift range and 15% zoom range, 720 images were generated, resulting in 1389 images used for training. A 5-fold cross-validation technique was used to avoid overfitting and to assess the model’s performance by evaluating it on five random subsets of the data (80% training, 20% validation). A holdout test set with 168 images was used for the test. The majority voting technique was used to identify the most frequently predicted class from the five folds, which was then selected as the final prediction. The batch size, indicating the number of training examples utilized in each training iteration, was set to 60. The number of epochs, representing how many times the complete training dataset would traverse the model during training, was set to 60. The learning rate was 0.0001.

#### 2.4.3. Evaluation Metrics

The performance metrics reported in this study were obtained using the hold-out test set. Four metrics were used for the evaluation of segmentation: the dice coefficient, which measures the similarity between the predicted segmented regions generated by the algorithm and the actual ground truth segmentation; the accuracy (1); the precision (2); and the sensitivity (3). The performance of a classification model is assessed using various metrics. The most widely used metrics are accuracy (1), precision (2), sensitivity (3), and F1-score (4) [5,20,21,22,23,24]. In our study, we employed these metrics to assess the classification task.

Accuracy = Number of Correctly Predicted Instances/Total Number of Instances(1)

Precision = True Positives/(True Positives + False Positives)(2)

Sensitivity = True Positives/(True Positives + False Negatives)(3)

F1-score = 2 × (Precision×sensitivity)/(Precision + sensitivity)(4)

A confusion matrix was used to represent the performance of the tested models. To illustrate the performance of the three classes on the VGG16-MLP model, the receiver operating characteristic (ROC) curve, representing the true-positive rate as a function of the false-positive rate, and the precision–recall curve were employed.

#### 2.4.4. Computational Setting

The experiments were conducted on 16 GB memory and a single NVIDIA GeForce GTX 1650 GPU. The CNN model was generated using the Keras library (https://keras.io accessed on 11 April 2024). The TensorFlow backend was used in this process (https://www.tensorflow.org accessed on 10 April 2024). Numpy library was applied for numerical computing (https://numpy.org accessed on 11 April 2024). OpenCV was employed for preprocessing and image analysis (https://opencv.org accessed on 11 April 2024) and Matplotlib was utilized for data visualization (https://matplotlib.org accessed on 11 April 2024).

### 2.5. Evaluation of Comparative Models

To compare the VGG16-MLP model’s performance in feature extraction, various pre-trained base models integrating a spatial attention mechanism with the two Depthwiseconv2D layers and combined with MLP were evaluated: VGG19-MLP, DenseNet121-MLP, DenseNet201-MLP, MobileNet-MLP, InceptionV3-MLP, and InceptionResNetV2-MLP. For the classification, the Support Vector Machine (SVM) classifier combined with the fine-tuned VGG16 model (VGG16-SVM) was evaluated and compared to VGG16-MLP.

### 2.6. Contribution Evaluation of Preprocessing, Spatial Attention Mechanism, and Depthwiseconv2D Layers

For VGG16-MLP model, the contributions of image segmentation, data augmentation, spatial attention mechanism, and Depthwiseconv2D layers were evaluated.

### 2.7. Visual Reader Analysis

A subjective visual analysis was performed by two blinded independent readers to classify healthy, MI, and myocarditis LGE images. The first reader was a radiology resident with 4 years of experience, and the second was a radiology resident with 3 years of experience.

### 2.8. Statistical Analysis

To describe the study population, the two-sample t-test was used to compare the age distribution between the different classes, and the chi-square test was used for the sex distribution. A Wilcoxon signed-rank test was used to compare the performance of VGG16-MLP with the other tested models, based on the accuracy values of the 5-fold cross-validation. The significance level was set at *p* < 0.05 for all tests. IBM SPSS version 26.0 was used for statistical analysis.

## 3. Results

### 3.1. Left Ventricle Segmentation

**Figure 3:**
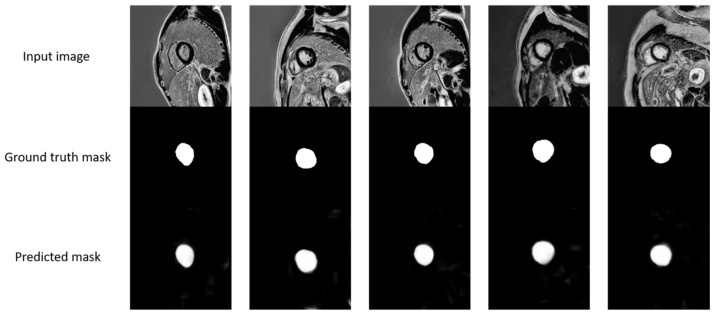
A visual representation of the input images, ground truth masks, and predicted masks.

**Figure 4:**
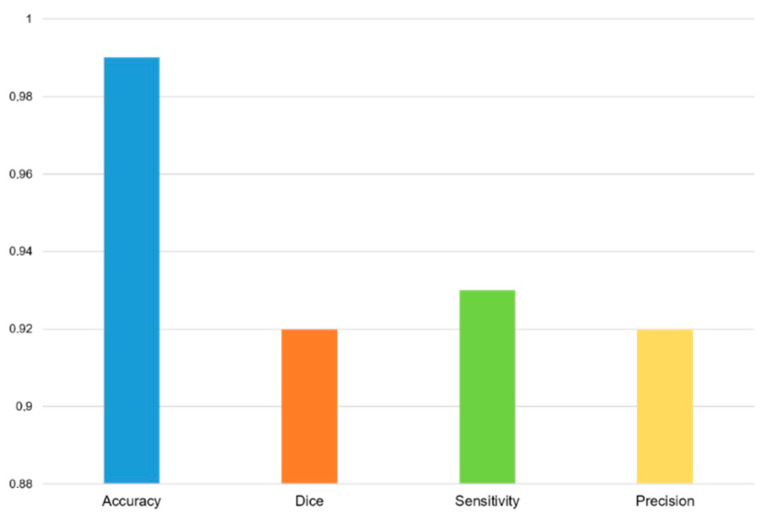
Bar charts of accuracy, dice coefficient, sensitivity, and precision of segmentation of the region of interest.

For the segmentation task, the dice coefficient was 92%, the accuracy was 99%, the sensitivity was 93%, and the precision was 92% (Figure 3 and Figure 4).

### 3.2. Performance of Models on Segmented Images

**Figure 5:**
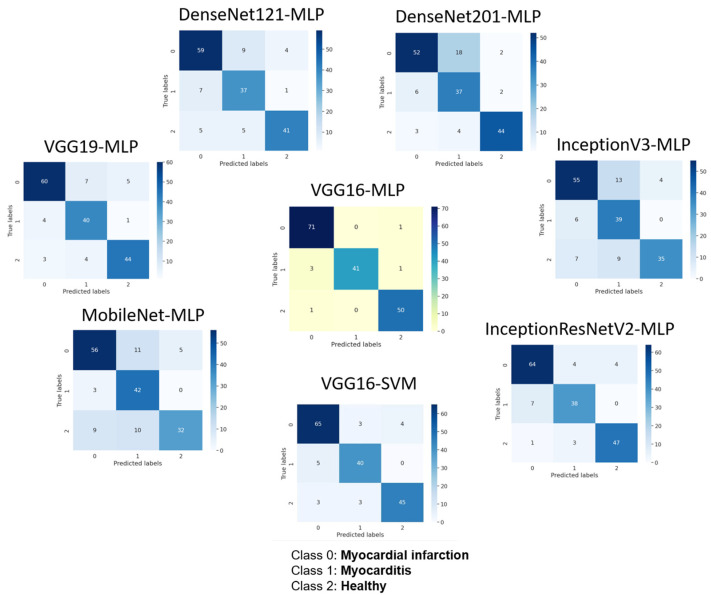
Confusion matrices of VGG16-MLP, VGG19-MLP, DenseNet121-MLP, DenseNet201-MLP, MobileNet-MLP, InceptionV3-MLP, InceptionResNetV2-MLP, and VGG16-SVM.

The accuracy, precision, sensitivity, and F1-score of VGG16-MLP, VGG19-MLP, DenseNet121-MLP, DenseNet201-MLP, MobileNet-MLP, InceptionV3-MLP, InceptionResNetV2-MLP, and VGG16-SVM are presented in Table 2. The VGG16-MLP model achieved the highest accuracy at 96%, precision at 97%, sensitivity at 96%, and F1-score at 96%. Most models demonstrated a statistically significant difference compared to VGG16-MLP (Table 2). The confusion matrices of tested models are presented in Figure 5. The training and inference times of the tested models (Table 2) demonstrated promising results, highlighting computational performance and feasibility for practical implementation.

#### VGG16-MLP Performance

**Figure 6:**
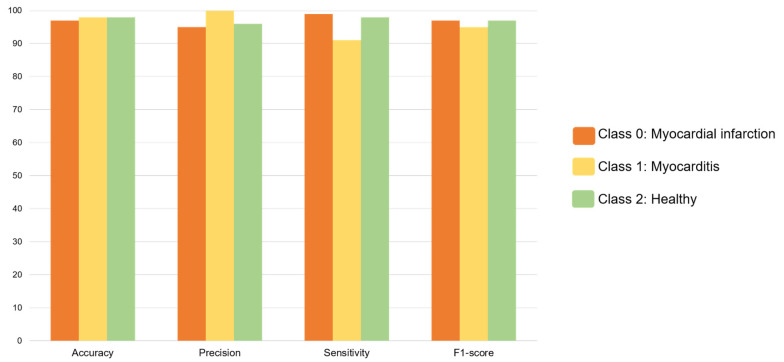
Bar charts of VGG16-MLP model’s accuracy, precision, sensitivity, and F1-score for myocardial infarction (class 0), myocarditis (class 1), and healthy (class 2). For the myocardial infarction, myocarditis, and healthy classes, the accuracy was 97%, 98%, and 98%; the precision was 95%, 100%, and 96%; the sensitivity was 99%, 91%, and 98%; and the F1-score was 97%, 95%, and 97%, respectively.

**Figure 7:**
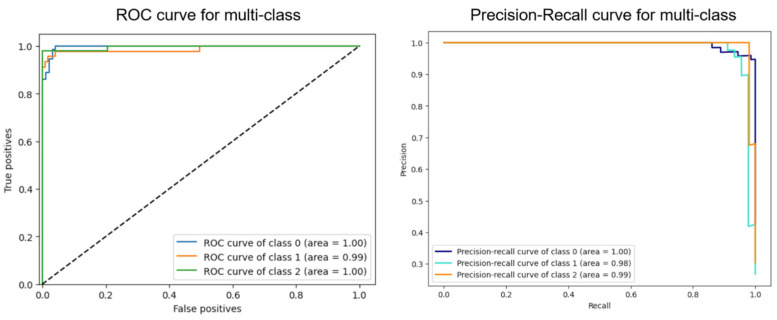
The receiver operating characteristic (ROC) curve and precision–recall curve of the myocardial infarction (class 0), myocarditis (class 1), and healthy (class 2) classes obtained from the VGG16-MLP model. In the ROC curves for the three classes, the true-positive rates are close to one, the false-positive rates are near zero, and the area under the curve (AUC) exceeds 0.9. The precision–recall curves indicate high values of precision and recall for the three classes, with AUCs of 1.00, 0.98, and 0.99 for the myocardial infarction, myocarditis, and healthy classes, respectively.

The VGG16-MLP model metrics for the MI, myocarditis, and healthy classes are presented in Table 3 and Figure 6. The accuracy reached 97% for MI, 98% for myocarditis, and 98% for the healthy class. The precision was 95% for MI, 100% for myocarditis, and 96% for the healthy class. The sensitivity was 99% for MI, 91% for myocarditis, and 98% for the healthy class. The F1-score achieved 97% for MI, 95% for myocarditis, and 97% for the healthy class. The confusion matrix is presented in Figure 5. The ROC curves (Figure 7) show the high performance of the three classes, as true positives are close to one and false positives are close to zero. The areas under the curve (AUCs) for the MI, myocarditis, and healthy classes were 1.00, 0.99, and 1.00, respectively. These values were superior to 0.9, which means that the diagnostic performance of the three classes was considered excellent [25]. The precision–recall curves (Figure 7) demonstrate high values of precision and recall for the three classes. The AUCs for the MI, myocarditis, and healthy classes were 1.00, 0.98, and 0.99, respectively.

### 3.3. Contribution Evaluation of Preprocessing, Spatial Attention Mechanism, and Depthwiseconv2D Layers

#### 3.3.1. Effect of Extracting the Region of Interest

For the VGG16-MLP model, the accuracy, precision, sensitivity, and F1-score of the original images before applying the segmentation were 87%, 89%, 86%, and 86%, respectively. So, in comparison to the segmented image results, the accuracy value increased by 9%, the precision by 8%, and the sensitivity and F1-score by 10% (Table 4).

#### 3.3.2. Effect of Data Augmentation

The accuracy, precision, sensitivity, and F1-score of our model without data augmentation were 86%, 86%, 87%, and 86%, respectively. Compared to the model results with data augmentation, the accuracy and F1-score increased by 10%, the precision by 11%, and the sensitivity by 9% (Table 4).

#### 3.3.3. Effect of Spatial Attention Mechanism

The accuracy, precision, sensitivity, and F1-score of VGG16-MLP without applying the spatial attention mechanism were 86%, 86%, 87%, and 86%, respectively. In comparison to the results using this function, the accuracy and F1-score increased by 10%, the precision by 11%, and the sensitivity by 9% (Table 4).

#### 3.3.4. Effect of Depthwiseconv2D Layers

The accuracy, precision, sensitivity, and F1-score of our model without applying the two layers of Depthwiseconv2D were 89%, 89%, 89%, and 88%, respectively. Compared to the model results using Depthwiseconv2D layers, the accuracy and sensitivity increased by 7%, and the precision and F1-score by 8% (Table 4).

### 3.4. Visual Reader Analysis

**Figure 8:**
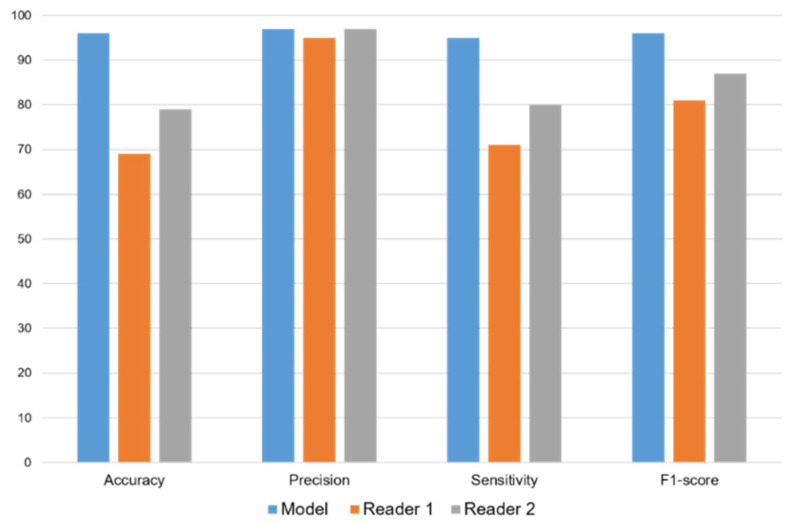
Bar charts of the accuracy, precision, sensitivity, and F1-score of our model VGG16-MLP, reader 1, and reader 2.

The first reader achieved an accuracy of 69%, precision of 95%, sensitivity of 71%, and F1-score of 81%. The second reader’s performance metrics were as follows: 79% accuracy, 97% precision, 80% sensitivity, and 87% F1-score. (Figure 8).

## 4. Discussion

CAD is expanding rapidly and is becoming widely used in radiological assessment [26]. The visual evaluation of MI, myocarditis, and healthy LGE images may be subjective and lead to incoherencies between readers. Hence, we decided to use deep learning to develop a CAD and enhance objectivity. Our study demonstrates that the VGG16-MLP model allows for distinguishing between MI, myocarditis, and healthy cardiac LGE images with a high accuracy level of 96% on automatically segmented images, which surpasses the two MRI readers. A recent study [5] aimed to determine if radiomics features extracted from LGE areas in cardiac MRI scans allow the differentiation between MI and myocarditis. This study was based on a machine learning algorithm. The highest accuracy was 89% using the SVM classifier. The classical medical image classification methods have not been sufficient to fulfill the current requirements due to the high complexity of medical images. In recent times, the evolution of deep learning theory has introduced a technical framework to answer the challenges of medical image classification [27]. According to our results, the use of deep learning improves the accuracy of the classification task and outperforms the subjective visual analysis. Deep learning architectures have the advantage of automatically extracting key features from datasets without explicit human intervention, as opposed to traditional machine learning, which requires experts’ manual selection of relevant features [11,12]. Another important advantage over machine learning is that it incorporates a greater number of learning layers and creates more advanced and generalized data representations through a superior level of abstraction [28]. In our study, VGG16-MLP was the best model for the classification of LGE cardiac MRI images compared to the other models combined with MLP (VGG19, DenseNet121, DenseNet201, MobileNet, InceptionV3, and InceptionResNetV2). The VGG16 model was introduced by K. Simonyan and A. Zisserman from Oxford University [18]. This architecture has gained considerable popularity in the research field [29] due to its simple structure of neural networks [23,29]. Thus, it has enhanced generalization capabilities and the ability to adapt to diverse datasets [18]. Several studies based on VGG16 for the classification task demonstrate the great performance of this model in various medical imaging modalities [16,18,24,30,31,32,33,34,35]. Tommaso et al. [5] evaluated the SVM model for the classification task of MI and myocarditis LGE images. In our work, SVM was assessed and compared with MLP for the classification task of MI, myocarditis, and healthy LGE images. The VGG16-MLP surpasses the VGG16-SVM in terms of accuracy, precision, sensitivity, and F1-score. It should be noted that image preprocessing is an important factor in improving the classification results. According to our study, segmenting the left ventricle significantly improves the accuracy of our model. Reducing unnecessary information in the images enables a more effective extraction of features. For instance, a study published in 2021 demonstrated that accuracy was higher when the image matrix was centered on the cardiac region of interest [16]. Furthermore, we used automatic segmentation using deep learning, which is characterized by greater consistency, reproducibility, and speed compared to manual segmentation, which is subjective and laborious. CNNs typically need to be trained on large numbers of images to achieve accurate performance [36]. To overcome the problem of limited data, we used the transfer learning technique first. It is the practice of developing high-performing learners who are pre-trained using readily available data from different domains [37]. It permits the exploitation of knowledge from the existing labeled data to improve a model’s performance with no labeled or limited data [38]. Secondly, we opted for the data augmentation technique. It is used to artificially expand the training set by performing small transformations on the existing data [39]. In our work, we applied a 15% zoom and a 10% horizontal shift range. We found that our model’s results were better using data augmentation. In fact, increasing the diversity of training data improves the robustness and performance of deep learning models, as well as their ability to generalize to the data [40]. To avoid overfitting and provide more reliable performance estimates, we used the K-fold cross-validation technique. It consists of dividing a dataset into k subsets; then, iteratively, some of them are used to learn the model, while others are used to evaluate its performance [41]. In addition to cross-validation, holdout test data were used. It allows the evaluation of the performance of our model on completely unseen data, which assesses its real-world efficacy on new data. It should be noted that deep learning faces challenges in medical image classification, including the difficulty of constructing high-performance models adapted to the characteristics of medical images and the limited adaptability of current network structures and training strategies. Thus, we used the spatial attention mechanism. In our work, this function considerably ameliorates the results of classification. Incorporating an attention mechanism into a deep learning model improves information extraction efficiency for medical image analysis and enhances the precision of reasoning [42]. Spatial attention can be seen as an adaptive mechanism for selecting spatial regions, inspired by human visual capabilities. This mechanism can be treated as a dynamic selection process for focusing on the most important features within an image [43]. Attention mechanisms integrated with CNNs were used in numerous studies for medical image classification [20,21,22,44]. The major limitation of our study is the limited dataset. Large datasets ensure greater performance for deep learning models. Secondly, other revealing images of myocarditis and MI, such as cine post-gadolinium and T2-weighted images, could be added to the dataset. Thirdly, we could consider including more types of cardiomyopathies in our model. Finally, in our study, all patients were included without exclusion during the data collection period, minimizing selection bias and ensuring a diverse representation of the clinical population. Additionally, all images were acquired using a single MRI scanner to reduce technical variability. The retrospective nature of the study may still introduce potential biases such as variations in image quality and patient demographics, which we will address by expanding the dataset. As a future perspective, our approach is to integrate clinical data into the algorithm to increase the diversity of the input data and thus improve the accuracy of the classification.

## 5. Conclusions

In conclusion, the VGG16-MLP model permits accurate classification of MI, myocarditis, and healthy LGE cardiac MRI images and could be considered a reliable CAD model. In order to enhance the robustness of our model, it would be advantageous to incorporate various datasets. The generalizability of our model to other cardiac diseases would require expanding the dataset with additional annotated data.

## Figures and Tables

**Table 1:** Dataset description.

	Healthy	MI	Myocarditis	*p* Value
Number of patients	27	89	47	
Age	42.25 (±14.62)	58.50 (±11.57)	29.06 (±10.19)	<0.001
Sex (F/M)	8/19	15/74	6/41	0.433
Number of images	254	361	222	
Total number of images		837		

**Table 2:** The accuracy, precision, sensitivity, F1-score, training time, and inference time of the tested models and the *p*-values of the statistical comparison of VGG16-MLP with other tested models.

Tested Models	Accuracy (%)	Precision (%)	Sensitivity (%)	F1-Score (%)	*p* Value	Training Time (s)	Inference Time (s)
VGG16-MLP	96 *	97 *	96 *	96 *	-	2372	0.738
VGG19-MLP	86	85	86	86	0.842	2921	0.954
DenseNet121-MLP	82	82	82	81	0.042 ^‡^	2450	0.606
DenseNet201-MLP	79	80	80	79	0.042 ^‡^	2481	0.870
MobileNet-MLP	77	79	78	77	0.043 ^‡^	2088	0.210
InceptionV3-MLP	77	78	77	77	0.043 ^‡^	2383	0.510
InceptionResNetV2-MLP	89	88	88	88	0.197	2610	0.984
VGG16-SVM	89	89	89	89	0.593	2422	0.702

* Highest performance. ^‡^: *p* < 0.05.

**Table 3:** VGG16-MLP model accuracy, precision, sensitivity, and F1-score for myocardial infarction, myocarditis, and healthy classes.

Classes	Accuracy (%)	Precision (%)	Sensitivity (%)	F1-Score (%)
Myocardial infarction	97	95	99	97
Myocarditis	98	100	91	95
Healthy	98	96	98	97

**Table 4:** Accuracy, precision, sensitivity, and F1-score of VGG16-MLP without image segmentation, data augmentation, spatial attention mechanism, or Depthwiseconv2D layers.

	Accuracy (%)	Precision (%)	Sensitivity (%)	F1-Score (%)
VGG16-MLP	96	97	96	96
VGG16-MLP without image segmentation	87	89	86	86
VGG16-MLP without data augmentation	86	86	87	86
VGG16-MLP without spatial attention mechanism	86	86	87	86
VGG16-MLP without Depthwiseconv2D layers	89	89	89	88

## Data Availability

Due to privacy and confidentiality concerns, the data supporting the findings of this study are not publicly available.
